# Early steps towards professional clinical note-taking in a Swedish study programme in dentistry

**DOI:** 10.1186/s12909-022-03727-7

**Published:** 2022-09-14

**Authors:** Nikolaos Christidis, Viveca Lindberg, Sofia Louca Jounger, Maria Christidis

**Affiliations:** 1 Department of Dental Medicine, Division of Oral Diagnostics and Rehabilitation, Karolinska Institutet, SE-141 04 Huddinge, Sweden; 2 Department of Teaching and Learning, Stockholm University, Stockholm, Sweden; 3 Department of Special Education, Stockholm University, Stockholm, Sweden; 4 Department of Health Sciences, The Swedish Red Cross University, SE-151 47 Huddinge, Sweden; 5 Department of Neurobiology, Care Sciences and Society, Karolinska Institutet, SE-141 83 Huddinge, Sweden

**Keywords:** Dentistry, Note-taking, Theory, Clinic, Undergraduate

## Abstract

**Background:**

Higher education tends to focus on academic writing only, instead of emphasizing that professional texts are also used as a basis for communication in contexts with a variety of participators. When it comes to clinical notes, research is scarce and focused on technology and informatics. Therefore, the aim was to explore dental students’ clinical notes, and specifically which aspects of the clinical notes characterizes clinical notes that are not sufficient enough for professional purposes.

**Methods:**

The object of analysis was the student’s written completion of a teacher constructed protocol regarding oral mucosa, the dental apparatus including pathology on tooth level, oral hygiene, and a validated international clinical examination protocol of the temporomandibular region. The study was framed within the New Literacy Studies approach, and the clinical notes were analyzed using thematic analysis.

**Results:**

Within the clinical notes three themes were identified; a) familiar content; b) familiar content in new context; and **c)** new content. The forms of notes could refer to either categorizational clinical notes or descriptive clinical notes. Most students were able to write acceptable clinical notes when the content was familiar, but as soon as the familiar content was in a new context the students had difficulties to write acceptable notes. When it comes to descriptive notes students suffered difficulties to write acceptable notes both when it came to familiar content, or familiar content in a new context.

**Conclusions:**

Taken together, the results indicate that students have difficulties writing acceptable notes when they are novices to the content or context, making their notes either insufficient, too short or even wrong for professional purposes. With this in mind, this study suggests that there is a need to strengthen the demands on sufficient professional quality in clinical notes and focus on clinical notes already in the early stages of the different medical educations.

## Background

The focus on literacy practices in context have become a promising theoretical basis for understanding that reading and writing are not only a question of functional literacy (the ability to read and write) but also a question of what and how people read and write in specific contexts, and for what purposes. Literacy practices are historically developed, maintained, and changed in relation to changes in context, for example scientific, technical, or societal. In a critical article by Moore and Morton (2017), the authors state that focus in several contemporary studies on professional literacy has been an emphasis on job-readiness and an ideological assumption that all higher education should prepare students for all aspects of professional knowing [1]. Higher education tends to focus on academic writing only, instead of emphasizing that professional texts are also used as a basis for communication in contexts with a variety of participators. Students need not only to become aware of but also to develop and become involved in this kind of text-based professional communication in order to become acquainted not only to the writing practices, but also to the communicative aspects of, that is, reading and oral communication related to written texts, and purposes for professional literacy, see also [2, 3].

According to Lea (2017), academic literacies must be understood in relation to contemporary changes in global higher education [4]. A previous focus on *disciplinary literacies* has moved towards vocational literacy, professional education and digitalization in terms of digital tools for learning and assessment. Furthermore, the globalization of the tertiary sector has changed what kinds of texts students are expected to produce [5]. Such studies have so far predominately been initiated within disciplines like anthropology, linguistics or educational sciences. Research that specifically focuses on clinical notes is scarce and the field seems to be dominated by researchers in technology and informatics. These studies have developed and tried out AI-applications where professional medical language in unstructured clinical notes has been translated to everyday language. They focus on 1) the need of patients to understand their doctors’ clinical notes [6], 2) challenges of working with clinical notes and benefits of developing automated machine learning for medical notes processing [7], 3) the use of machine learning for professional purposes, and 4) to learn deep representation of patient notes with the specific purpose of identifying high-risk readmission to medical care [8, 9].

Insufficient or incorrect clinical notes risk to place patients in a vulnerable position. This, since clinical notes are the base for clinical reasoning, diagnostics, treatment plan and for the outcome of the patient treatment or even mistreatment [10]. Therefore, it is understandable why clinical note-taking is such an essential part in patient care. Also, they are not just a source of information for health professionals but also for the patients. Further, it has been indicated that undergraduate health professions students need training in medical record keeping. However, it has also been shown that these students are graded for their clinical observations rather than their literacy skills [11]. Since clinical notes prepare for professional dental record keeping, we argue, in line with Schreyer (2003, p. 204) that dental record keeping is a central discursive practice that needs to be further investigated.

With this in mind, to explore the characteristics of students’ clinical notes is a field of research that needs attention as part of the knowing needed for becoming a member in a community of praxis [12], in this particular case, a dentist. The aim of this study was therefore to explore dental students’ clinical notes and specifically to which aspects of the clinical notes characterizes clinical notes that are not sufficient enough for professional purposes. Since these notes are not only for personal use but also the basis for communication with dental colleagues as well as other health care professions, such notes need to adhere to a predetermined structure as well as to a content. This needs to be related to dental terminology, but also to specific ways of expressing oneself in writing. Furthermore, we also have an interventionist interest since we specifically want to discern what knowing and experience teaching needs to address for an acceptable result. This, since our theoretical points of departure assumes that teaching and learning are reciprocal [13, 14].

## Methods

### Context of the study

To analyze the student-aspect of clinical note-taking, during a clinical examination of the masticatory system including an analysis of the occlusal aspects is based on clinical protocols used in the undergraduate Study Programme in Dentistry (SPD). The clinical protocols were collected between January and March 2018 at the University Dental Clinic, Department of Dental Medicine, Karolinska Institutet (KI). The students involved in this study were in their third year of the SPD. Twenty-four clinical protocols (in total 120 pages), by 24 students working in pairs, were subject to a thematic analysis. The students who wished to volunteer and to contribute with clinical notes for analysis of note-taking were informed to first remove any kind of identifications (i.e. name) from the protocols and to give a verbal informed consent at two occasions, first to the principal investigator (NCh) and then to an external researcher (MCh). The students were instructed by the external researcher (MCh) to check that they had removed all possible identifications before handing over the protocols. After receiving the protocols, the external researcher (MCh) scanned all protocols pairwise and renamed them Protocol 1 to 12, in order to keep anonymity. Thus, no identifications were possible to track on the digital files since these files only had the credentials of the external researcher. The students were prepared for the clinical part of the course with in total eight lectures (45 minutes each) during two semesters, some of them during the preceding semester and the others during the same semester as the examination (for a specification of content, see [15]). The students were on their third year of the SPD at KI and participated in the module “Orofacial Pain and Jaw Function 2”, part of the course “Clinical Odontology 2” (https://utbildning.ki.se/course-syllabus/2TL016/24160). For further information of the structure of the SPD in relation to this study, this has been described in detail in Christidis and co-workers (2021). Prior to this module, the students have had training in communication with patients at basic level of dental education, e.g. in semesters 1 and 2 in the course “To become a dentist 1, 7.5 credits”. They have also had training in reading and writing patient records at basic level of dental education, e.g. in semesters 1 and 2 in the course “To become a dentist 1, 7.5 credits”, and in semesters 3 and 4 in the course “Clinical odontology 1, 24.5 credits” – especially in a) Module 1: Patient documentation and introduction to the clinic, 1.4 credits, but also in b) Module 3: Periodontology, 7.6 credits; c) Module 4 Cariology, 5.2 credits, and d) Module 6 Integrated clinical dentistry in adults, 1.2 credit.

### Object of analysis

The object of analysis was the student’s written completion of: **1)** a teacher constructed protocol regarding oral mucosa, the dental apparatus including pathology on tooth level, oral hygiene, i.e. this part of the protocol has been taught in previous courses, while the following part regarding analysis of the occlusion and jaw-relations was new to the students; **2)** an international clinical examination protocol of the temporomandibular region (i.e. the masticatory system), for both clinicians and researchers, according to the Diagnostic Criteria for Temporomandibular Disorders (DC/TMD) [16], followed by an evaluation of the use of an occlusal appliance (i.e. bite-splint) which also is teacher constructed, these were also new to the students*.* Preceding this clinical task, the students participated in two three-hour lectures as well as a clinical demonstration by two teachers (SLJ, NCh) regarding the analysis of the occlusion and jaw-relations and examination of the temporomandibular region. This can be seen as a simulation task since students work in pairs. However, they were expected to complete the protocols individually. As a note from the two teachers (SLJ, NCh), some of the students took over the answering by filling in their fellow student’s protocol.

**Fig. 1:**
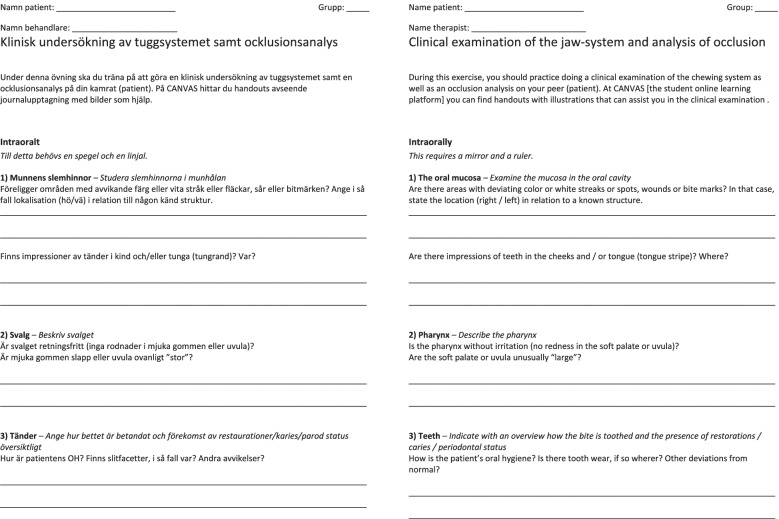
Illustration of the teacher constructed protocol and its translation to English (by the authors)

For the teacher constructed protocol the students are expected to describe the oral mucosa, the pharynx and the teeth. If there are variations to normal mucosa or pathological findings in the oral mucosa including the hard palate, these should be described concerning shape, size, color, location; if there are impressions in the cheeks or tongue also the degree of keratinization should be described in addition to shape, size, color, and location. Regarding the pharynx they are asked to describe if there are any signs of inflammation or irritation both in the pharynx and the soft palate. When it comes to the teeth, the students are asked to describe the dentition, i.e., which teeth are present, if they have signs of caries, periodontal disease, which restorations are present including their quality, degree of tooth wear or if there are any deviations from normal, exemplified in Fig. 1. Finally, there is a part with an occlusal analysis (i.e., how the teeth are in contact when biting and moving the jaw), similar to the example in Fig. 1.

**Fig. 2:**
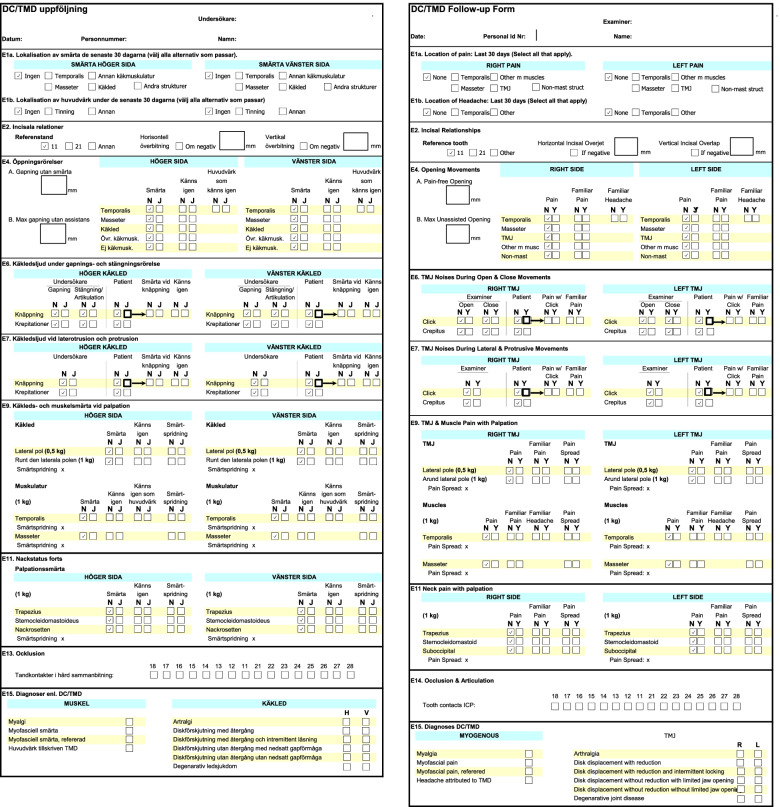
Illustration of the validated, standardized clinical examination protocol, according to the diagnostic criteria for temporomandibular disorders both in Swedish and English

Regarding the clinical protocol according to DC/TMD, the students are just expected to assess the jaw opening, check the occlusion i.e., the relation between teeth upon biting, and to fill in boxes with “Yes” or “No” regarding pain upon movement and/or palpation of the joints, jaw- and neck muscles, Fig. 2. When it comes to the evaluation of the use and effect of the occlusal appliance, they are expected to describe the occlusal appliance, i.e., the degree of wear, fitting, and appliance hygiene. After that, they are expected to ask the patient, in this case their clinical partner, about their experience and summarize the patient experience in words, similar to the example in Fig. 1.

### Theoretical framework and analysis

Since dental students’ notetaking is considered a social practice [17], New Literacy Studies (NLS) can be used as the framework for the analysis of this study [18, 19]. NLS is the overarching theoretical framework, whereas we used a thematic analysis of data as a first step. This combination gave us access to patterns and helped us discern meaning, a summary, an interpretation that attempted to theorize the importance of the patterns, as well as their general meanings and inferences within a specific theoretical context, in our case NLS [20, 21].

Central for NLS is that *literacy* is considered as social practices, which means that *what* and *how* people read and write, as well as the *purpose(s)* for reading and writing differs between contexts. Entering a professional higher education programme means that students also enter new literacy practices. On the one hand, they are introduced to the *academic dental literacy practice*, on the other, they are also introduced to and gradually appropriate a *dental professional literacy practice*. During their studies, they are expected to be able to shift between these two literacy practices, depending on the purpose of the texts they read and write [22]. Furthermore, such texts are written for communicative purposes – there is an assumption that the writer should match the need of a future reader, making communication possible over time and between contexts that results in a predictable interpretation of the text by the reader [1, 23].

In order to find patterns in data, different methods for identifying, analyzing and reporting patterns were used. In this material consisting of students’ clinical notetaking thematic analysis was chosen since this method supports interpretation concerning different aspects of the topic of study involving a rich description of data, and a minimal organization of data [21, 24]. In this study, a theme is determined by what it captures in relation to clinical notetaking. Thus, based on the clinical notes from the 24 volunteering students, themes, relating to the data, were created. These themes are ultimately related to the study’s research questions and were created by a thorough, inclusive, and comprehensive coding process, including interpreted data and not data paraphrased or described. Each theme has been described in detail and given a nuanced account [20, 21, 24]. Thus, the thematic analytical procedure involved noticing patterns of meaning and issues of potential interest in the data. Analysis also involved a relational reading, which in this case was a constant moving back and forth between the protocols and the analysis of data produced. The analytical steps performed in relation to clinical notes of 24 dental students were as follows (Braun & Clarke 2021, p. 331): 1) data familiarization and writing familiarizations notes; 2) systematic data coding for each student in relation to the following analytical question: what content is noted vs what is not expressed in the notes?; 3) generating initial themes from coded and collated data (step 2); 4) developing and reviewing themes in relation to the identified questions and to the entire data set. This control ensures that the set themes are internally coherent, consistent and distinctive; 5) refining, defining and naming themes. The themes were: (i) Familiar knowledge, (ii) New knowledge, and (iii) insufficient distinction between anamnesis and status; and 6) writing the report and selecting extract examples that illustrate the themes. Finally, the selected extracts were related back to the research question of the study to ensure coherence. Familiar knowledge relates to knowledge that the student has acquired from previous courses. New knowledge, on the other hand, relates to knowledge acquired from current and ongoing courses.

## Results

Based on the students’ notes, three themes were identified. These were a) familiar content; b) familiar content in new context; and c) new content, shown in Table 1. The forms of notes could refer to either categorizational clinical notes or descriptive clinical notes as visualized in Table 1. As presented in the methods-section the categorizational clinical notes could for instance concern presence or absence of caries or periodontitis etc., tooth-naming, checking boxes for yes/no or just adding measurements, etc. Further, when it came to the descriptive clinical notes the students were supposed to interpret what they saw in the patient’s mouth and translate it to an illustrative and informative text. When analyzing the content in the clinical notes written by the students, they were classified as either *acceptable* (marked with “√” in Table 1) with regards to academic and professional demands, or *not acceptable* (marked with “– “in Table 1). *Acceptable* in terms of academic demands includes both theoretical learning outcomes and clinical skills according to both the national as well as the international protocols. *Acceptable* in terms of professional demands includes notes that should be sufficient, adequate, and communicative to dental colleagues or, eventually, also to other health care professions. This difference guided us to ask what patterns are common in each of these types of clinical notes, so the thematic analyses were done separately for each of these types.

**Table 1:** Common patterns identified in clinical notes from 24 dental students

Content in clinical notes	Forms of notes			
	Categorizational clinical notes	Degree of familiarity	Descriptive clinical notes	Degree of familiarity
***Oral mucosa***	✓ (*n* = 19)	Familiar content	- (*n* = 12)	Familiar content
***Pharynx***	✓ (*n* = 16)	Familiar content	- (*n* = 9)	Familiar content New context
***Teeth***				
Present/absent	✓ (*n* = 24)	Familiar content	*na*	*na*
Caries	✓ (*n* = 24)	Familiar content	*na*	*na*
Periodontal status	✓ (*n* = 24)	Familiar content	*na*	*na*
Oral hygiene	*na*	*na*	✓ (n = 24)	Familiar content
Tooth wear	✓ (*n* = 24)	Familiar content	*na*	*na*
Restaurations	✓ (*n* = 24)	Familiar content	✓ (*n* = 24)	Familiar content
***Dental relations***	- (*n* = 8)	New content	*na*	*na*
***Occlusion***				
Static	✓ (*n* = 17)	Familiar content	- (*n* = 2)	Familiar content New context
Dynamic	- (*n* = 5)	Familiar content New context	- (*n* = 2)	Familiar content New context
***DC/TMD protocol***	✓ (*n* = 23)	New content	*na*	*na*
***History taking***	*na*	*na*	- (*n* = 8)	Familiar content New context
***Evaluation of treatment***	*na*	*na*	- (*n* = 5)	Familiar content New context

*na* Not applicable, *DC/TMD* Diagnostic Criteria for Temporomandibular Disorders

### Categorizational clinical notes

Most students were able to write acceptable clinical notes when the content was familiar, but as soon as the familiar content was in a new context (occlusal analysis) the students had difficulties to write acceptable notes. This is illustrated by the students’ answers in Example A (author translated).

Example A: Normally, you have a well-defined IP [intercuspid position] that can undoubtedly be found, which means that free habitual closing movements moves the lower jaw directly to the IP, and with only minor muscle power. When the patient is sitting comfortably and relaxed upright (90 °) in the clinical examination chair with the gaze straight ahead, can she or he then take the IP as above?

***Student 1 answer***: “– “[a line, that is, no information].

*Author comment:* In this case, this line makes it difficult for others to interpret whether the student did not check this item or if there were no relevant findings. Here the information is professionally insufficient, demanding a supplementary short clarification.

***Student 2 answer***: “u.a.” [the English equivalent abbreviation is n.a.d., meaning nothing abnormal detected].

*Author comment:* This note was found representing professionally insufficient information for other dentists as well as other professionals to interpret.

Note, for this and the following examples, the terms used mean the following: “Example” is the instruction for the task, “Student answer” is the students clinical note for the task, and “Author comment” is a description of our interpretation and an explanation on why the “Student answer” is acceptable or not.

When new content was presented, students were able to correctly check yes/no boxes (in the DC/TMD protocol, Fig. 2), but when they had to interpret what they saw in the patient’s mouth and translate it to a status/patient record (regarding dental relations) they were not able to correctly translate their findings based on clear criteria written in the protocol (example of criteria written in the protocol presented in Example B), as shown in the students’ answers in Example B (author translated).

Example B: Dental relations – Describe the dental relations from a vertical. Measure the vertical and horizontal overjet.

***Vertical aspect.***

**Frontal view:** Look at the vertical overjet on [tooth number] 11, 21.

*Normal bite* **= 1–4** mm overjet.

*Open bite* **≥ − 1** mm overjet.

*Edge-to-edge bite* **= 0** mm overjet.

*Deep bite* **> 4** mm overjet.

**Lateral view:** Look in the side areas, is there contact between the teeth or is there air in between?

**Vertical overjet:** ____ mm.

***Frontal view*** *:* normal bite ☐ open bite ☐ edge-to-edge bite ☐ deep bite ☐.

***Lateral view*** *:* normal bite ☐ open bite right ☐ open bite left ☐.

***Student 3 answer***: “Vertical overjet 4.5 mm => normal bite”. *More that 4 equals deep bite.*

***Student 4 answer***: “Vertical overjet 0.5 mm => normal bite”. *Less than 1 equals edge-to-edge bite.*

*Author comment:* Both students do not seem to understand that there are specific criteria for each dental relation, but instead they interpret them as approximal.

### Descriptive clinical notes

When it comes to descriptive notes students suffered difficulties to write acceptable notes both when it came to familiar content (Example C1 and C2), or familiar content in a new context (Example D). In this module there was no new content that the students needed to describe in their clinical notes:

Example C1: Oral mucosa part 1 - Study the mucosa of the oral cavity. Are there areas with deviating color or white streaks or spots, wounds or bite marks? In that case, state the location (right / left) in relation to any known structure.

***Student 5 answer***: “– “[a line, that is, no information].

*Author comment:* In this case (as in Example A), this line makes it difficult for others to interpret whether the student did not check this item or if there were no relevant findings. Here the information is professionally insufficient, demanding a supplementary short clarification*.*

***Student 6 answer***: “u.a.” [the English equivalent abbreviation is n.a.d., meaning nothing abnormal detected].

*Author comment:* This note was found representing professionally insufficient information for other dentists as well as other professionals to interpret (as in Example A).

Example C2: Oral mucosa part 2 - Are there impressions of teeth in the cheeks and/or the tongue (tongue stripe)? Where?

***Student 7 answer***: “Yes”.

*Author comment:* This student’s answers to the question shows that (s)he can distinguish between normal and deviating mucosa (i.e. categorize), but she cannot describe it in terms of localization or appearance.

Example D: If the occlusal contacts do not coincide, what can be the consequences for the muscle activity during chewing?

***Student 8 answer***: “– “[a line, that is, no information].

*Author comment:* In this case (as in Example A and C), this line makes it difficult for others to interpret whether the student did not check this item or if there were no relevant findings. Here the information is professionally insufficient, demanding a supplementary short clarification.

***Student 9 answer***: “u.a.” [the English equivalent abbreviation is n.a.d., meaning nothing abnormal detected].

*Author comment:* This note was found representing professionally insufficient information for other dentists as well as other professionals to interpret (as in Example A and C).

***Student 10 answer***: “Yes and No?”

*Author comment:* The total of this student’s answers to the questions in this part of the protocol shows that (s)he cannot distinguish between normal and deviating dental relationships, one of the basic abilities for a dentist. This indicated by the question marks‚ and by answering both yes and no to the question.

## Discussion

The main findings from this study were that three different themes of clinical notes could be identified. The dental students either write clinical notes based on a) a familiar content in a *familiar* context, or b) a familiar content in a *new* context, or c) new content. The thematic analysis could also show that the clinical notes could be either categorizational or descriptive. Most students were able to write acceptable clinical notes when the content was familiar and in a familiar context, but as soon as the familiar content was in a new context the students had difficulties to write acceptable notes. When it comes to descriptive notes students had difficulties to write acceptable notes both when it came to familiar content, or familiar content in a new context. This would result in insufficient, too short or even clinical notes carrying wrong information, from a professional point of view. The consequence of inadequate information exchange, which may place primary care patients in a vulnerable and exposed situation, with risk of not being able to ensure the quality of care and the patient safety [9, 25]. Even in other health professions it has been shown that high-quality clinical notes, i.e. patient documentation, is crucial and that the quality of the clinical notes/patient documentation is inadequate, thus placing patients in a great risk [25–28]. One can only speculate, why the clinical notes are inadequate. Hypothetically, this can be explained by insufficient knowledge, but it can also be explained as insufficient guidance in note-taking practice [29]. However, this material cannot provide answer to this.

In cases where students had experiences of both identifying e.g. caries and writing clinical notes, we found that the notes were rich and sufficiently informative for professional purposes. In cases where students instead were novices to the physiological and pathological examples, their notes became either insufficient, too short or even wrong for professional purposes. This is a problem often discussed as an aspect of transfer from one situation, in this case academic writing, to another, in this case professional writing. This has also been found in other health professions where for instance registered nurses experience lack of skills, knowledge, and even confidence necessary for documentation tasks. This, although they had already received both an undergraduate education and formal training on the topic [25]. Säljö (2003) offers another interpretation of the problem: that it is rather a question of crossing a boundary from one context to another [30]. In this case it would mean that students need to be made aware of firstly, the differences in literacy demands within academia and the profession, and secondly, to be able to identify the new situation as *a situation where a specific knowing is needed*. The results of the study by Theo et al. (2021) strengthen the need for focusing on the demands on sufficient professional quality in clinical notes already during dental education.

Another interesting finding was that, during examination, the fellow-students took over the answering for the patients by e.g. filling in what they thought the patient was expressing. The consequence of this is that students did not realize the assignment in accordance with the intentions, since the fellow-students do not adopt a patient-role that is expected in the simulation. One explanation to this may be because the assignment was a simulation, and the patients were fellow students which also are difficult to act professionally with [31]. Other negative aspects raised regarding simulation assignments are nervousness in simulation situation and the use of a “dummy’s speech” [31, 32]. Despite these negative aspects, the clinical simulation settings prepare the students for the professional setting [33].

A final finding was that it seems that it was unclear for the students that the courses within the study programme build upon each other and that there is an expected progression in the study programme. They are therefore expected to combine previous knowledge with new knowledge. The research material showed that students had a difficulty in discriminating between normal, deviant/variation of normal and pathological conditions but also to discern the new knowledge, that is, the aspects in a fellow student’s mouth. Difficulties in discerning new knowledge, certain aspects related to professional knowing, is common in other professions too, as shown by for instance Meek in 2005 [29] who advocates for *authoritative guiding* to support students in learning to see, that is to discern what the professional eye needs to see. In this case, the discerning is one aspect of the knowing needed, while the professional form for, as well as the demands on adequate documentation discussed above is the other. Thus, as Bruner stated already in 1973 scientific thinking is impossible when you are not able to go “beyond the information given “ [34]. In a medical professional situation that is to be able to distance yourself from the actual text, i.e. clinical notes/patient documentation, and to look at the phenomenon or condition described in the text. To be able to do this the students as well as the different health professionals need to learn and train to integrate new knowledge with previous knowledge, in order to achieve the skill to differentiate between what is said by the patient or found in the examination and what they previously know [35].

## Conclusions

Taken together, the results indicate that students have difficulties writing acceptable notes when they are novices to the content or context, making their notes either insufficient, too short or even wrong for professional purposes. The consequences of inadequate clinicals notes will result in inadequate information exchange, which may place patients in a vulnerable and exposed situation, as well as in a great risk of mistreatment.

With this in mind, this study suggests that there is a need to strengthen the demands on sufficient professional quality in clinical notes, and to focus on clinical notes already in the early stages of the different medical educations, using for instance authoritative guiding.

## Data Availability

The materials used and analyzed during the current study are available from the corresponding author on reasonable request.

## Abbreviations

DC/TMD: Diagnostic Criteria for Temporomandibular Disorders
KI: Karolinska Institutet
na: Not applicable
NLS: New Literacy Studies
SFS: Svensk Författningssamling
SPD: Study Programme in Dentistry
